# The hyperexcitability of laterodorsal tegmentum cholinergic neurons accompanies adverse behavioral and cognitive outcomes of prenatal stress

**DOI:** 10.1038/s41598-023-33016-2

**Published:** 2023-04-12

**Authors:** Mohammad Shabani, Mehran Ilaghi, Reyhaneh Naderi, Moazamehosadat Razavinasab

**Affiliations:** 1 Intracellular Recording Lab, Neuroscience Research Center, Neuropharmacology Institute, Kerman University of Medical Sciences, P.O. Box 76198-13159, Kerman, Iran; 2 Laboratory of Emotions Neurobiology, Nencki Institute of Experimental Biology, Polish Academy of Sciences, Pasteur Street 3, 02-093 Warsaw, Poland; 3 Department of Physiology, Kerman University of Medical Sciences, Kerman, Iran

**Keywords:** Developmental biology, Neuroscience

## Abstract

Exposure to prenatal stress (PS) leads to the offspring's vulnerability towards the development of cognitive and behavioral disorders. Laterodorsal tegmentum (LDT) is a part of the brainstem cholinergic system that is believed to play a pivotal role in the stress-associated progression of anxiety, memory impairment, and addictive behaviors. In this study, we aimed to investigate the electrophysiological alterations of LDT cholinergic neurons and its accompanied behavioral and cognitive outcomes in the offspring of mice exposed to physical or psychological PS. Swiss Webster mice were exposed to physical or psychological stress on the tenth day of gestation. Ex vivo investigations in LDT brain slices of adolescent male offspring were performed to evaluate the effects of two stressor types on the activity of cholinergic neurons. Open field test, elevated plus maze, passive avoidance test, and conditioned place preference were conducted to assess behavioral and cognitive alterations in the offspring. The offspring of both physical and psychological PS-exposed mice exhibited increased locomotor activity, anxiety-like behavior, memory impairment, and preference to morphine. In both early- and late-firing cholinergic neurons of the LDT, stressed groups demonstrated higher firing frequency, lower adaptation ratio, decreased action potential threshold, and therefore increased excitability compared to the control group. The findings of the present study suggest that the hyperexcitability of the cholinergic neurons of LDT might be involved in the development of PS-associated anxiety-like behaviors, drug seeking, and memory impairment.

## Introduction

Prenatal stress (PS) is generally referred to as the stress, both physical or psychological, experienced by a mother during pregnancy. Accumulating clinical and animal studies have demonstrated that PS raises the risk of neurodevelopmental disorders in later life periods in the offspring. For instance, offspring born to mothers who experienced stress during pregnancy have been shown to demonstrate higher anxiety levels, depression-like behaviors, and cognitive disorders in the adolescence and adulthood^1–4^. In addition, it has been shown that stressful events during pregnancy could affect the vulnerability of the offspring to addiction and drug abuse in later life^5,6^. Cognitive dysfunctions, including the persistence of aversive memories^7,8^, and impaired spatial learning and memory^9–11^ of the offspring, are other significant consequences of PS.

Various mechanisms have been put forth as to how PS could promote the incidence and development of psychiatric disorders in newborn. The hypothalamic–pituitary–adrenal (HPA) axis is the main stress response pathway, activated through the stimulation of stress hormones' release, which include corticotropin-releasing factor (CRF), adrenocorticotrophic hormone (ACTH), and cortisol (or corticosterone in rodents)^12^. On the other hand, chronic stress alters acetylcholine (Ach) release in different regions of the brain and the hyperactivity of the cholinergic system is known to be associated with the development of psychiatric disorders, including anxiety, depression and substance use disorder (SUD)^13,14^.

The laterodorsal tegmentum (LDT) nucleus is located in the pontine central gray, beneath the caudal part of the aqueduct, and connects the third and fourth ventricles. The LDT contains predominantly cholinergic, glutamatergic, and GABAergic neurons and is known as a part of brainstem cholinergic system^15^. It has been indicated that the LDT contains CRF- and urocortin (Ucn1)-containing neurons^16–18^, which are involved in the stress-associated progression of anxiety, depression, SUD, inappropriate arousal, and control of the sleep and wakefulness^19–23^. These neurons contribute to the elevated risk of psychiatric disorders linked to chronic stress by modulating the cholinergic transmission of LDT^24^. In addition, LDT is involved in controlling mood through cholinergic excitatory projections to the ventral tegmental area (VTA), which is a fundamental regulator of emotional behaviors^25–27^. Furthermore, LDT activates VTA dopaminergic neurons via glutamatergic and cholinergic pathways, increasing the dopamine (DA) release in the nucleus accumbens (NAc)^28,29^. Therefore, it is not surprising that LDT plays an important role in drug-dependent behaviors, as previous studies have indicated that this nucleus is associated with drug-seeking behaviors and increased risk of addiction induced by amphetamine, nicotine, and morphine^30–32^.

**Figure 1:**
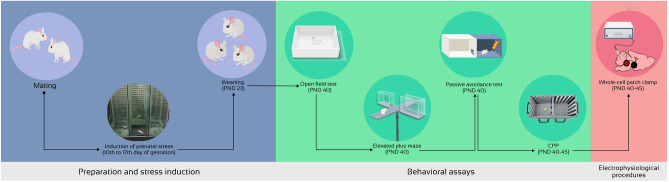
Timeline of prenatal stress induction, behavioral assays and electrophysiological procedures.

Previously, we have reported that prenatal exposure to both physical and psychological stress during fetal development changes drug-seeking and anxiety-like behaviors in the offspring, which is associated with the hyperexcitability of VTA dopaminergic neurons^33^. These neurons receive inputs from LDT and are tightly modulated by cholinergic projections. LDT-VTA projections are vital for the activity of dopaminergic neurons in the VTA and control DA-related behaviors^28,31^. Based on these findings, the present study was carried out to examine the effects of physical and psychological stress on the electrophysiological properties of LDT cholinergic neurons in Swiss Webster mice. In addition, changes in passive avoidance learning and memory, locomotor activity, anxiety-like behavior and addiction were examined in offspring (Fig. 1).

## Results

### Effect of PS on locomotor activity and anxiety-like behavior of the offspring

**Figure 2:**
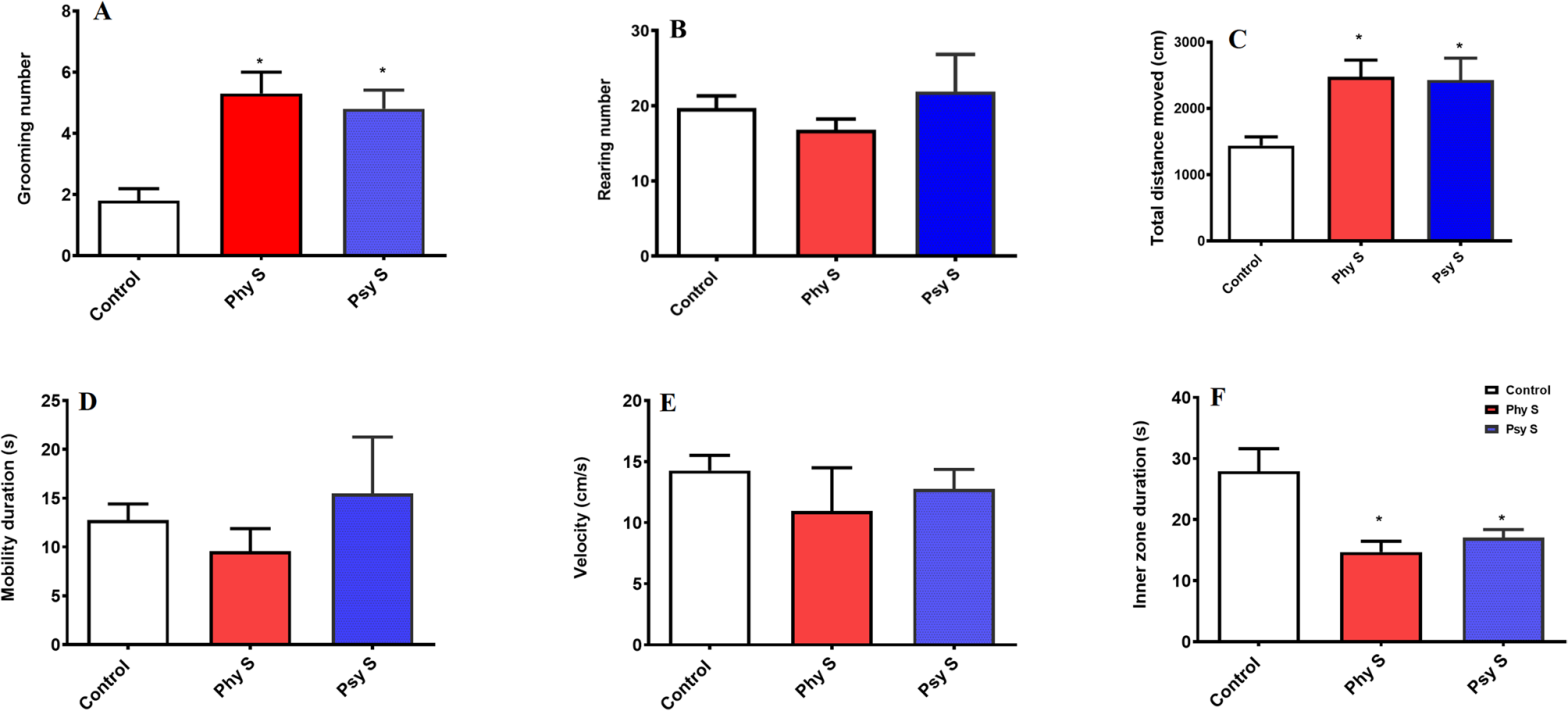
Comparison of the effect of PS on locomotor activity and anxiety-like behavior of the offspring in the open field test. (**A**) Frequency of grooming per session. (**B**) Frequency of rearing per session. (**C**) Total distance moved. (**D**) Duration of mobility. (**E**) Mean velocity. (**F**) Time spent in the center zone. Data are displayed as Mean ± SEM. Phy s: physical stress, Psy s: psychological stress, * (*p* < 0.05), ** (*p* < 0.01), *** (*p* < 0.001) compared to the control group.

In order to evaluate the locomotor activity and anxiety-like behavior, the total time spent in the center zone, total distance moved, mobility duration, velocity, and the number of grooming and rearing were assessed in the open field test (Fig. 2).

The number of grooming in the offspring of dams exposed to physical and psychological stress was significantly increased compared to the control group (F _2, 27_ = 2.34; *p* = 0.014, Fig. 2A). However, no significant difference was observed in the number of rearing between the groups (Fig. 2B).

The total distance moved (F _2, 27_ = 7.2; *p* = 0.003) in the two groups of physical stress (*p* < 0.01) and psychological stress (*p* < 0.001) was significantly higher compared to the control group (Fig. 2C). No significant difference was observed in the mobility duration among the groups (F _2, 27_ = 0.6; *p* = 0.54, Fig. 2D). In terms of anxiety-like behavior, there was no significant difference in movement velocity between the study groups (F_2, 27_ = 0.4; *p* = 0.6, Fig. 2E), however, time spent in the center zone was significantly lower in the two prenatally stressed groups compared to the control group (F_2, 27_ = 5.4; *p* = 0.018, Fig. 2F).

**Figure 3:**
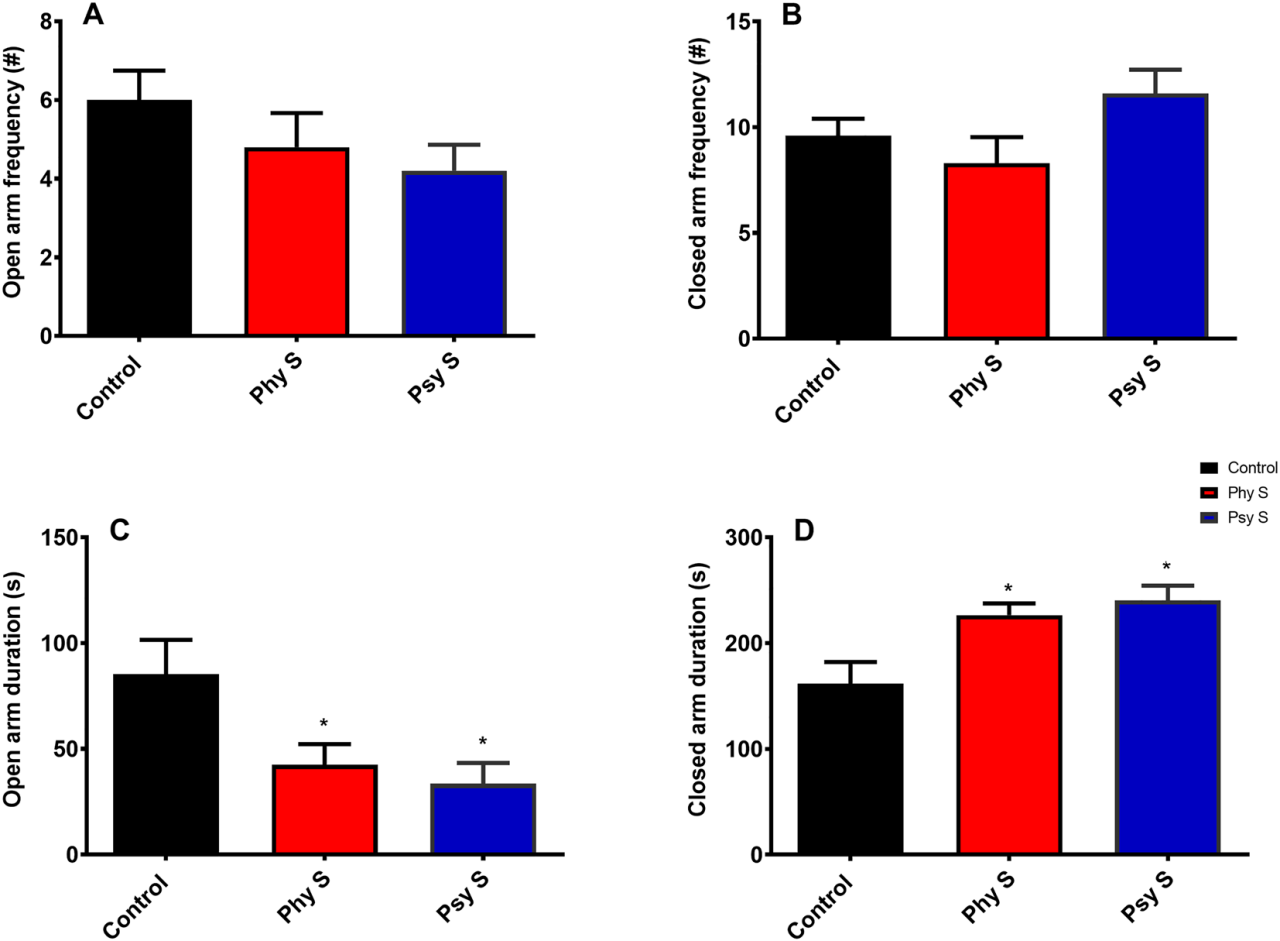
Assessment of the effect of PS on anxiety-like behavior of the offspring in EPM. (**A**, **B**) Frequency of entries into open and closed arms. (**C**, **D**) Time spent in open and closed arms. Data are displayed as Mean ± SEM. Phy s: physical stress, Psy s: psychological stress, * (*p* < 0.05) compared to the control group.

The EPM test revealed that there was no significant difference in terms of the frequency of entries into open and closed arms between any of the groups (Fig. 3A, B). However, the offspring of both physical and psychological PS-exposed groups, demonstrated a significant shorter time spent in the open arms compared to the control group (F _2, 27_ = 6.1; *p* = 0.013, Fig. 3C). Accordingly, both stressed groups had significantly spent more time in the closed arms in comparison to the control group (F _2, 27_ = 3.5; *p* = 0.02, Fig. 3D).

### Effect of PS on passive avoidance learning and memory of the offspring

**Figure 4:**
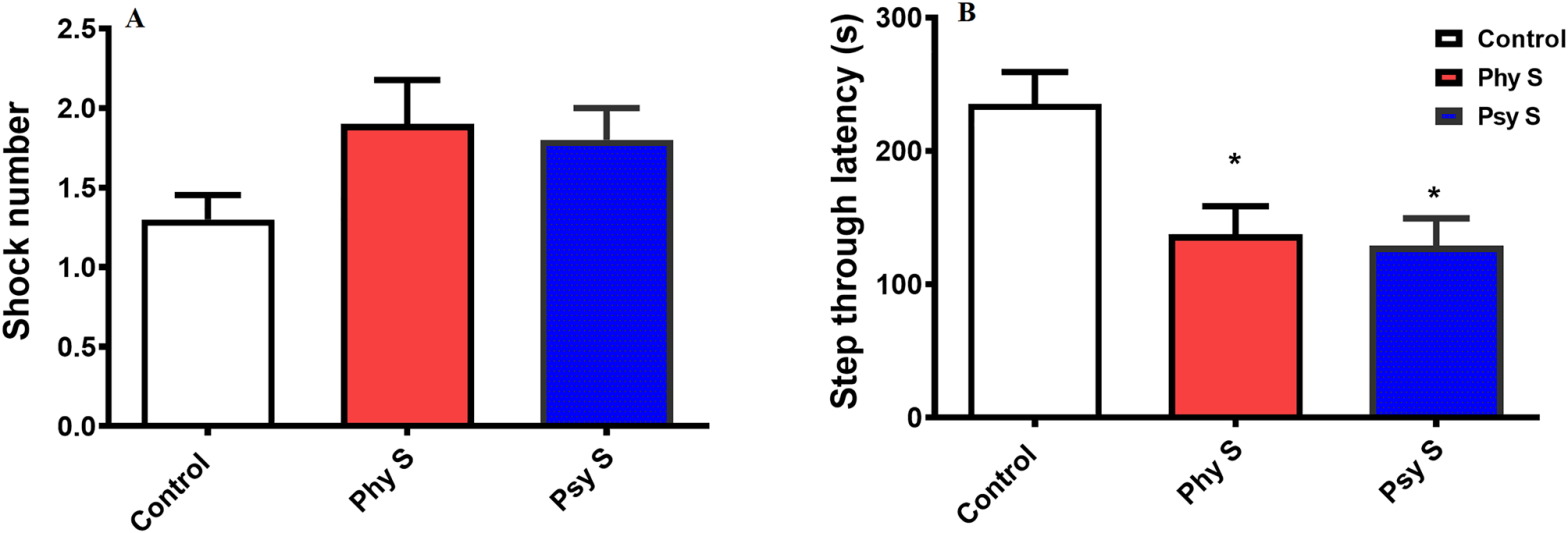
The effect of PS on passive avoidance learning and memory of the offspring. (**A**) Number of shocks required for learning. (**B**) Step through latency. Data are displayed as Mean ± SEM. Phy s: physical stress, Psy s: psychological stress, * (*p* < 0.05) compared to the control group.

In the passive avoidance memory test using shuttle-box, no difference was observed between the prenatally stressed and the control group in terms of the number of shocks (F _2, 27_ = 2.2; *p* = 0.12, Fig. 4A), indicating that the stress applied did not affect avoidance learning. On the other hand, a significant decrease in STL was observed in both the physically and psychologically stressed groups compared to the control group (F_2, 27_ = 7.2; *p* = 0.019, Fig. 4-B), demonstrating that stress, despite not affecting learning, impaired the memory of mice exposed to prenatal physical and psychological stress.

### Effect of PS on morphine-induced place preference of the offspring

**Figure 5:**
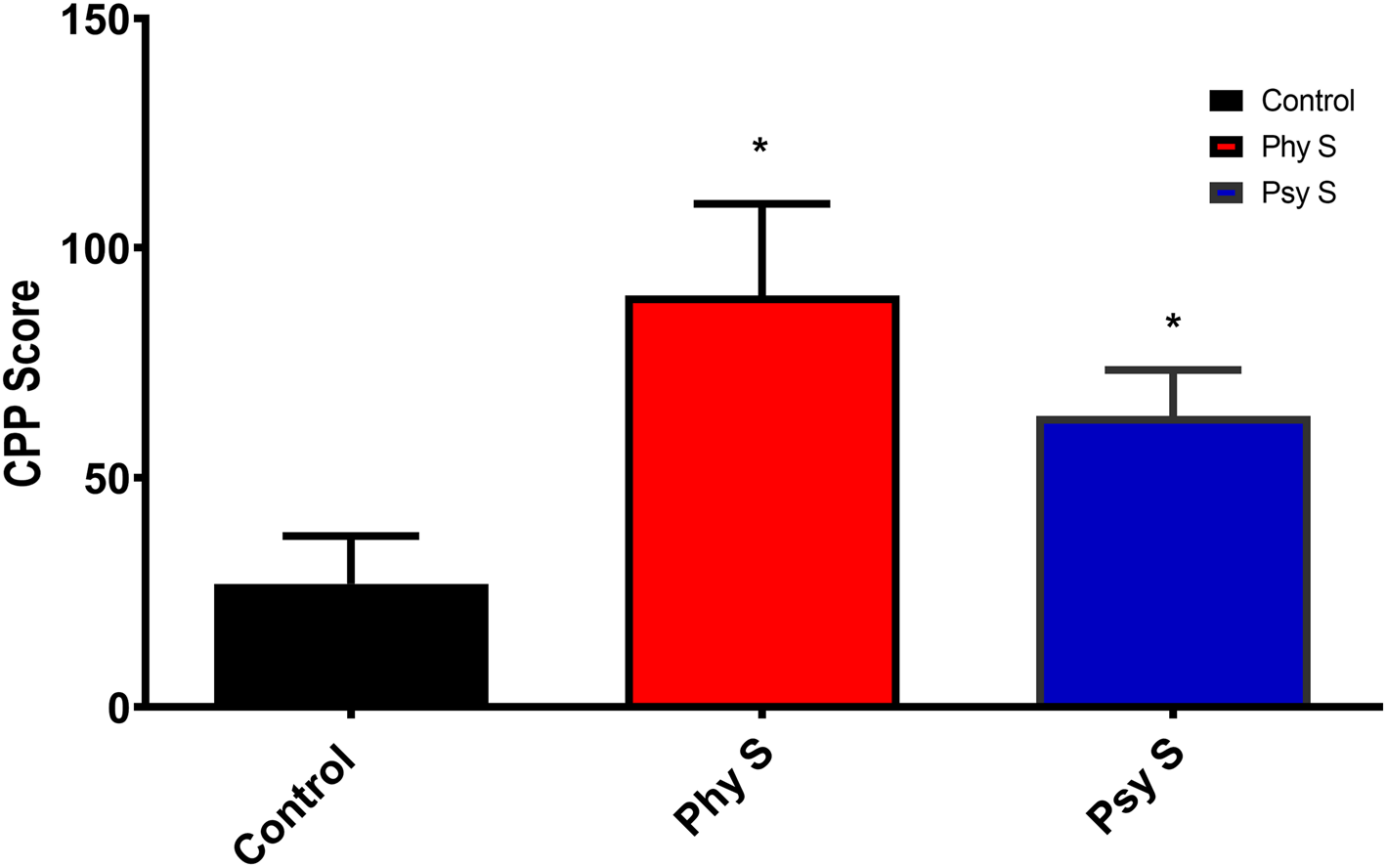
Assessment of the effect of PS on conditioned place preference (CPP) score of the offspring. CPP score is defined as the difference in time spent in the non-preferred chamber on post-conditioning day and the time spent in the non-preferred chamber on pre-conditioning day. Data are displayed as Mean ± SEM. Phy s: physical stress, Psy s: psychological stress, * (*p* < 0.05) compared to the control group.

Analyzing the CPP score in the offspring of both stressed groups demonstrated a higher preference for the morphine-paired compartment compared to the control group (F _2, 27_ = 3.7, *p* = 0.03; Fig. 5), indicating that physical and psychological PS, results in an increased preference for morphine in the offspring.

### Recording of acetyl cholinergic neurons of lateral dorsal tegmentum

No significant differences were observed between the groups in terms of the passive properties of the LTD cell membranes. The results are shown as membrane resting potential, internal resistance, series resistance and membrane capacitance in Table 1.

**Table 1:** Passive properties of laterodorsal tegmentum cell membranes.

LDT	Control	Phy S	Psy S	*P*-value
RMP (mV)	− 63 ± 4.2	− 61 ± 3.8	− 59 ± 4.5	NS
R_in_ (MΩ)	115.3 ± 11	102 ± 9	98 ± 14	NS
Ra (MΩ)	13 ± 1.2	12 ± 0.6	13 ± 1.4	NS
Cm (pf)	38.5 ± 3.2	41 ± 5.2	45 ± 6.3	NS

RMP (mV): Resting membrane potential (mV), R_in_ (MΩ): Internal resistance (megaohm), Ra (MΩ): Series resistance (megaohm), and Cm (pF): membrane capacitance (picofarad). NS: Non-significant (*p* > 0.05). Data are represented as Mean ± SEM. Phy S: Physical stress, Psy S: Psychological stress.

**Figure 6:**
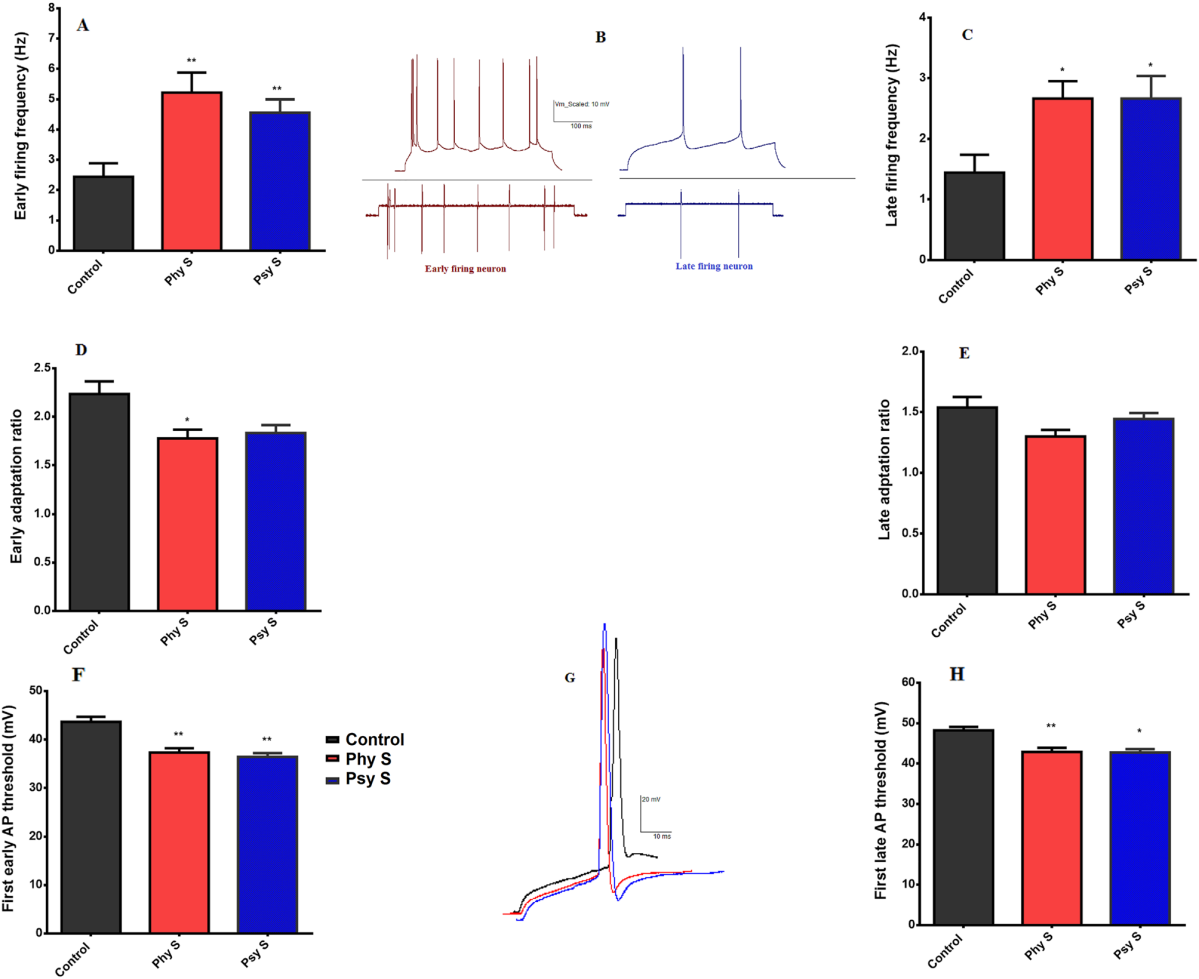
Injection of 100 pA positive current for 500 ms after clamping at − 70 Mv in offspring of physical and psychological prenatal stressed mothers. (**A**) Firing frequency of early-firing neurons. (B) Firing pattern of early- and late- firing neurons. (**C**) Firing frequency of late-firing neurons. (**D**) Adaptation ratio of early-firing neurons. (**E**) Adaptation ratio of late-firing neurons. (**F**) AP threshold of early-firing neurons. (**G**) AP pattern of early- and late- firing neurons. (**H**) AP threshold of late-firing neurons. Data are represented as Mean ± SEM. * (*p* < 0.05), ** (*p* < 0.01) shows a significant difference compared to the control group. Phy S: Physical stress, Psy S: Psychological stress.

To determine the electrophysiological properties of cholinergic neurons, 100 pA positive current was injected for 500 ms, while the cell was held at a negative 70 mV voltage, and the cell response voltage was recorded. The first group of identified cells, consisted of neurons that responded rapidly, with the first spike spaced less than 42 ms apart. The second group of cells, exhibited less adaptation ratio and the manifestation of first spike in response to the positive injection current was more than 75 ms apart (Fig. 6B).

In response to a positive current of 100 pA in mice born to physical and psychological PS-exposed mothers, the frequency of spikes was significantly higher compared to the control group, both in early-firing (F _2, 24_ = 7.2, *p* = 0.002) and late firing (F _2, 24_ = 4.8, *p* = 0.017) neurons (Fig. 6A, C).

Compared to the control group, the offspring of physical PS-exposed mice, showed a significantly lower adaptation ratio in early-firing neurons (F _2, 24_ = 5.8, *p* = 0.018). However, no significant difference was observed in the late-firing neurons (F _2, 24_ = 3.3, *p* = 0.06; Fig. 6-D, E).

Pups born to physical and psychological PS-exposed mothers had significant decreased action potential (AP) threshold compared to control group, both in early- and late-firing neurons [(F _2, 24_ = 18.1, *p* = 0.002); (F _2,24_ = 11.2, *p* = 0.018); Fig. 6F, G, and H]. The alteration of electrophysiological properties in the two groups of neurons, was recorded in response to 100 pA positive current injection. In terms of the number of APs and the first AP latency**,** a significant difference was observed within the stressed and control groups, both in early- and late-firing neurons (Table 2).

**Table 2:** Electrophysiological properties of early- and late-firing neurons.

		Action potential (#)	AHP (mv)	First AP latency	First AP amplitude (mV)	First AP half-width (ms)
Control	**Early-firing neurons**	2.4 ± 0.4 *P* = 0.003** F(2,24) = 9.2	10 ± 0.9 *P* = 0.17 F(2,24) = 3.4	29 ± 2.7 *P* = 0.004** F(2,24) = 8.2	66.6 ± 1.3 *P* = 0.2 F(2,24) = 1.42	1.8 ± 0.06 *P* = 0.3 F(2,24) = 1.1
	**Late-firing neurons**	1.4 ± 0.2 *P* = 0.009** F(2,24) = 6.4	15.2 ± 09 *P* = 0.5 F(2,24) = 0.8	135.3 ± 12.2 *P* = 0.02* F(2,24) = 6.8	66.3 ± 1.1 *P* = 0.2 F(2,24) = 1.7	2 ± 0.07 *P* = 0.2 F(2,24) = 1.4
Physical stress	**Early-firing neurons**	5.2 ± 0.6	12.2 ± 0.5	19.4 ± 0.9	64.8 ± 1.1	1.7 ± 0.04
	**Late-firing neurons**	2.6 ± 0.2	16.6 ± 0.9	101.9 ± 4.2	64.6 ± 0.5	1.8 ± 0.08
Psychological stress	**Early-firing neurons**	4.5 ± 0.4	12.4 ± 0.5	21.5 ± 1.6	64.2 ± 1.1	1.7 ± 0.04
	**Late-firing neurons**	2.6 ± 0.4	16.3 ± 0.7	102.2 ± 5.5	64.3 ± 0.7	1.8 ± 0.09

AHP = Afterhyperpolarization. Data are represented as Mean ± SEM. * (*p* < 0.05), ** (*p* < 0.01) within groups.

Significant values are in bold.

**Figure 7:**
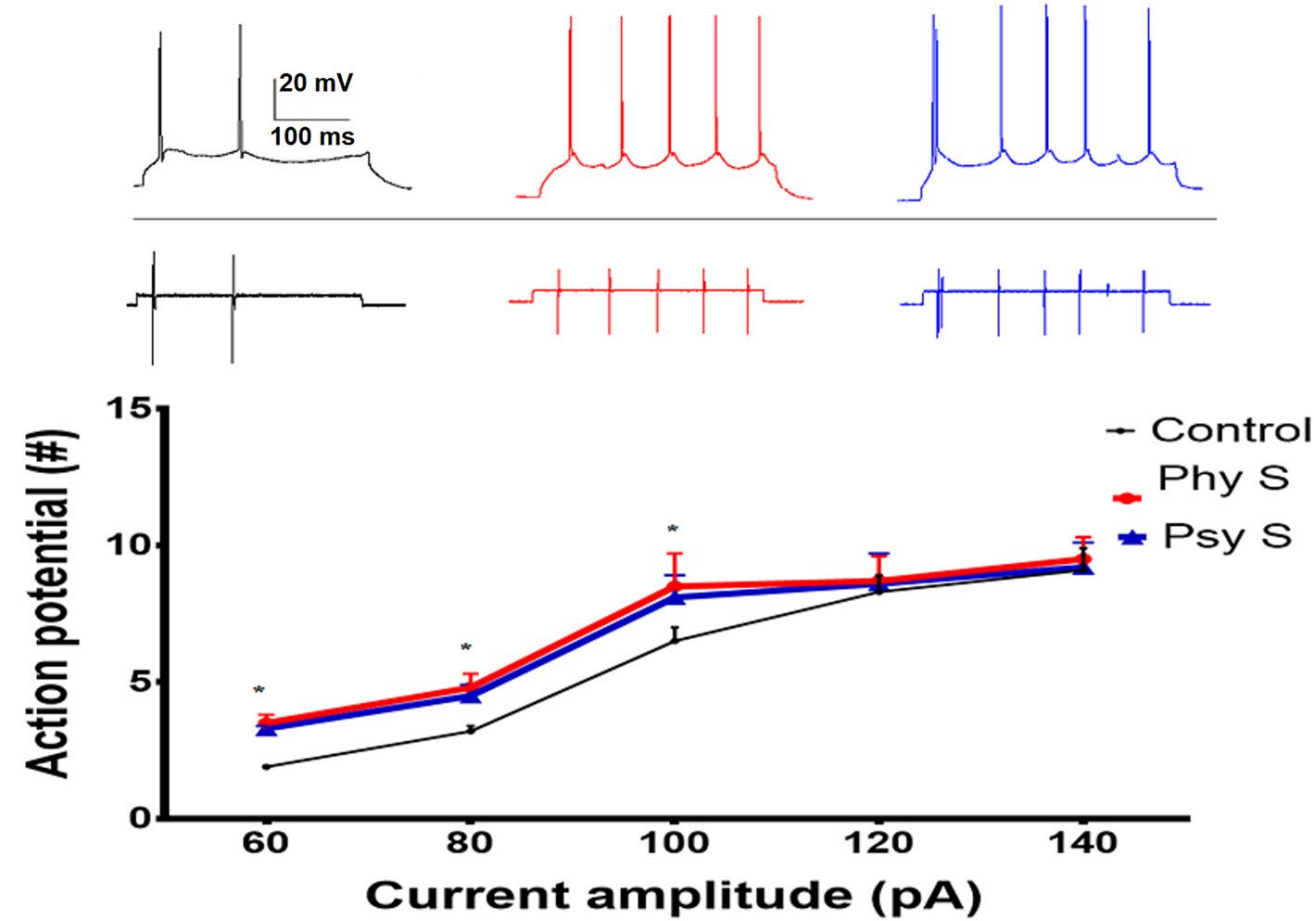
Early-firing neurons' response pattern to positive current injection pulses with intensities of 60 to 140 pA. Data are represented as Mean ± SEM. * (*p* < 0.05) showed a significant difference compared to the control group. Phy S: Physical stress, Psy S: Psychological stress.

Additionally, the response of early-firing neurons to positive current injection pulses was recorded at intervals of 500 ms with intensities of 60 to 140 pA at a 20 pA increasing pace. Only at the lowest stimulation intensity of 60 pA, neurons in both PS groups showed a significant increase in the number of APs compared to the control group (Fig. 7). Using the same protocol in late-firing neurons, no significant difference was observed between any of the groups.

**Figure 8:**
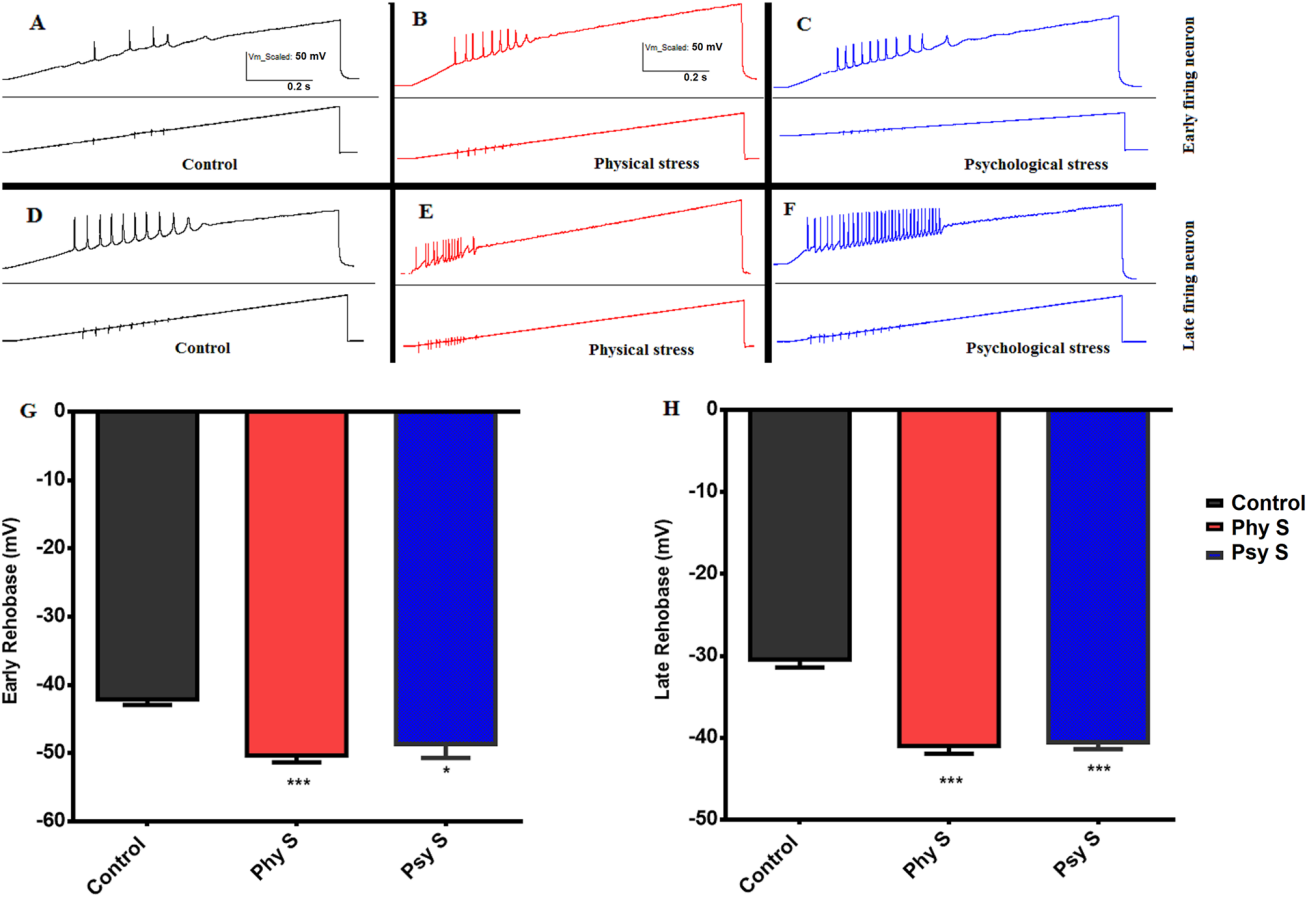
Response of early-firing neurons (**A**–**C**) and late-firing neurons (**D**, **F**) to Ramp flow injection pulses (0 pA to 2nA lasting 1000 ms) and their corresponding early (**G**) and late (**H**) rheobase. Data are represented as Mean ± SEM. *** (*p* < 0.001) * (*p* < 0.05) shows a significant difference compared to the control group. Phy S: Physical stress, Psy S: Psychological stress.

In order to evaluate the degree of excitability and rheobase activity of early- and late-firing neurons, injection of an increasing ramp current (0 pA to 2nA lasting 1000 ms) was implied in both early-firing (Fig. 8A–C) and late-firing neurons (Fig. 8D, F) of the control and stressed groups. The results demonstrated that prenatal physical and psychological stress decreases the voltage required to reach the threshold and firing AP and increases the excitability in both cell types [(F _2, 24_ = 42.6, *p* = 0.0008); (F _2, 24_ = 9.2, *p* = 0.013); Fig. 8-G-H). In other words, mice with stress conditions in both cell types were stimulated at more negative voltages and had lower thresholds than the control group.

## Discussion

Clinical and animal studies have demonstrated that PS can lead to aberrant alterations in the behavior and physiological function of the offspring. In our previous study, we showed that the hyperexcitability of VTA dopaminergic neurons of mice whose dams were exposed to prenatal physical or psychological stress was associated with alterations in drug dependency and anxiety-like behaviors through mesocortical and mesoaccumbal pathways^33^. The LDT-VTA pathway plays a critical role in regulating the activity of VTA DA neurons and the development of motivated behaviors^28,31^. It has been indicated that LDT regulates stress through CRF- and Ucn1-containing neurons^16–18^, which are involved in the development of stress-associated disorders, including SUD, inappropriate arousal, anxiety, and depression^19–21^. The high activity of cholinergic neurotransmission has also been shown to be involved in the progression of psychiatric disorders^34,35^. Interestingly, stress-related neuropeptides exert neuronal excitation in LDT cholinergic neurons, which are associated with an increased risk of psychiatric disorders^24^. In addition, it has been shown that cognitive functions are extremely dependent on the cholinergic system, where deficiency in cholinergic neurotransmission results in disturbances in learning and memory^36,37^. Pharmacological studies have also demonstrated that cholinergic antagonists induce amnesia in experimental subjects^38,39^. In light of these findings, the present study was designed to evaluate the effects of PS stressor type on synaptic and membrane properties of LDT cholinergic neurons in addition to the changes in the motivated behaviors of the offspring.

Our findings demonstrated that LDT cholinergic neurons were differentiated into two subtypes, early- and late-firing neurons, based on their firing delays in response to depolarizing current injections. Early-firing neurons are more excitable and have prominent spike frequency adaptation, while late-firing neurons are less excitable and maintain a tonic firing pattern at low frequencies^40^. We further highlighted the role of LDT cholinergic transmission in PS-associated behavioral outcomes. Our novel findings showed that PS leads to the hyperexcitability of both early- and late-firing cholinergic neurons of the LDT by increasing the firing frequency and decreasing the voltage required to reach the threshold and firing AP. Previous evidence has shown that LDT stimulates the DA neurons in the VTA, increasing the amount of DA released in the NAc^26,28,31^. Interestingly, it has been reported that PS affects the expression of DA receptors and the electrophysiological activity of DA neurons in NAc and VTA, respectively^33,41^. Moreover, PS or prenatal exposure to high levels of glucocorticoids (GCs) induce long-lasting changes in the expression of choline acetyltransferase (ChAT) and acetylcholine esterase (AChE) as cholinergic markers in the LDT. These molecular changes in the LDT-VTA circuit, which accompany prominent motivational deficits, could be rescued by optogenetic activation of the LDT-VTA terminals^42^. Therefore, LDT may be involved in mood control and addictive behavior through the regulation of DA release in mesolimbic regions. According to these findings, our results raise the possibility that physical and psychological PS may lead to the hyperexcitability of LDT cholinergic neurons, which further results in alterations in the LDT-VTA pathway and the regulation of DA.

We also demonstrated that pups born to physical and psychological PS-exposed mice exhibited a significantly increased tendency to morphine compared to the control group. This finding underscores the importance of stressors during fetal development on the incidence of addiction in the offspring. As previously indicated, there is growing evidence reporting that offspring born to PS-exposed mothers elicit a predisposition to dependency on various types of drugs, including cocaine^43^, amphetamines^44^, and morphine^45^. In reward-related circuits, stress-related neurotransmitters and neuromodulators, such as CRF, the κ-opioid agonist dynorphin, and noradrenaline (NA), contribute to addictive behaviors^46–49^. Neurobiological studies have shown that LDT is involved in the dependency on a wide range of drugs of abuse^30–32^. Based on these findings, LDT might play a pivotal role in mediating the effects of PS on the drug-seeking behavior of the offspring.

Studies have indicated that both physical and psychological stress during pregnancy increase the risk of depression and anxiety in the newborn^50,51^. In this regard, we have previously reported that exposure to physical and psychological stress during fetal development produces anxiety-like behavior in offspring, which is accompanied by the hyperexcitability of dopaminergic neurons in the VTA^33^. Similarly, our findings in this study revealed that both stressor types resulted in increased locomotion and anxiety-like behavior of the offspring. In line with previous studies, we further demonstrated that these stressors resulted in impaired passive memory of the offspring as well^52^. Building on this evidence, we propose that behavioral and memory deficits due to PS are accompanied by the hyperexcitability of cholinergic neurons in LDT. Therefore, in addition to VTA, cholinergic neurons of LDT might be other pieces of the puzzle contributing to the mechanisms underlying the behavioral outcomes of PS in the offspring.

## Conclusion

Overall, the findings of the present study demonstrated that exposure to physical or psychological stress during fetal development elicits behavioral and cognitive deficits in the offspring, which are accompanied by the hyperexcitability of LDT cholinergic neurons. Future endeavors could provide further explanation on how the VTA-LDT circuit is implicated in the outcomes of PS. This may offer novel therapeutic targets aiming to reduce the burden of PS on the offspring, therefore ameliorating the resulting behavioral and cognitive disorders.

## Material and methods

### Subjects

Swiss Webster mice were prepared from the Kerman University of Medical Sciences. The animals were caged in a photo period-controlled room (12-h light/dark cycle) with a temperature of 25 ± 2 °C, and prior to the introduction of the male, females were housed in cages of three. Food and water were available ad libitum. All experimental procedures were approved by the Animal Research Ethics Committee of the Kerman Neuroscience Research Center (Ethics code: IR.KMU.REC.1397.579). Successful mating was verified by the detection of a vaginal plug and pregnant females were randomly assigned to the control, physical stress, and psychological stress groups (n = 10 in each group), and housed two per cage.

### Drugs

Salts that have been used to prepare the intra- and extracellular solutions were purchased from Sigma-Aldrich (St. Louis, MO).

### Induction of prenatal stress

A previously established method was used to confront pregnant animals with physical or psychological stress. The stress procedure commenced on tenth day of gestation and continued for the next 7 days using the following protocol^52,53^. The Communication Box, a 3 × 3 glass house aquarium (25 × 25 × 60 cm^3^ in volume) containing 5 + 4 cells (nine cells in total), was used to expose animals to stressors. The cells were separated from each other by transparent Plexiglas, which allowed mice in different cells to see one another. Cells housing the pregnant dams exposed to physical stress were connected to an electric pump that filled the cells with water (20 ± 2 °C). Animals were placed in the houses of the aquarium that were filled with water, and animals were forced to swim in the water for 5 min. Afterward, the house drained and the animals could rest for 10 min. Subsequently, the house was filled with water again. All parts of procedure lasted for 1 h and animals were then dried and moved to their cages.

The mice in the psychological stress group were placed in the houses of aquarium that were not connected to the pump and just observed the other animals receiving the physical stress of swimming. Control mice were brought to the laboratory and remained there for the same duration as stress-exposed groups.

Pups were delivered, and the number of offspring, mortality rate and birth weight were recorded for each animal. Two male offspring were selected from each litter and were weaned from their mothers on the 23rd day and were then caged in groups of four (n = 20 for each group; 10 for behavioral tests and 10 for electrophysiological assays). As several previous studies have demonstrated that male and female offspring are differentially affected by prenatal stress^52,54,55^, to avoid any potential conflicting results that might be attributed to hormonal changes seen in the female mice, in the current study we used males only. The behavioral tests and electrophysiological assays were performed on postnatal days (PND) 40–45. Figure 1 provides an overview of the study's timeline (Fig. 1).

### Behavioral procedures

The following behavioral tests were conducted in order on each group of offspring mice with a 15-min rest period in between the tests:

#### Open field test

The apparatus consisted of an arena made of opaque Plexiglas (90 × 90 × 45 [H] cm). The arena was divided into central and peripheral regions, which contained 16 squares. Each mouse was placed in the center of the arena. The horizontal and vertical activities were recorded for 5 min, and were analyzed with offline analysis (Ethovision7.1, Noldus Information Technology, The Netherlands). Total time spent in the center or periphery, total distance moved, mobility duration, velocity, and the number of grooming and rearing were recorded for each animal. At the end of each trial, the maze was cleaned with a cotton cloth and 70% ethanol^56^.

#### Elevated plus maze (EPM)

The EPM was made of wood and was located 38 cm above the ground. The maze consisted of two open arms and two closed arms, each 50 cm in length. The illumination at the center was adjusted to 100 lx (red light)^57,58^. The animals were placed individually at the center of the platform facing one of the open arms and allowed to move freely on the maze for 5 min. During the test, the percentage of the time spent in open arms and the number of entries into the open arms were recorded using a video camera installed above the maze. The apparatus was carefully cleaned up with cotton and alcohol between the tests.

#### Passive avoidance test

A shuttle-box apparatus was used to evaluate associative learning and memory, which consisted of one lighted and one dark chamber. The two chambers were separated by a grid door, and a shock generator was linked to the floor rods of the dark chamber^56^. Before the test, the mice were placed individually in the light arena of the apparatus. After 10 s, the door was opened and the animal was allowed to go to the dark arena without electric shock for 30 s. One hour later, the acquisition phase was performed, and each mouse was placed into the light chamber. The door was then opened and the animal was allowed to enter the dark compartment. The animal received an electrical shock (0.5 mA, 50 Hz) upon entrance to the dark chamber via the stainless steel rods for 2 s. This part was repeated up to five times at 30 min intervals until the learning of the animal to avoid entering the dark compartment occurred. The number of shocks required for learning to happen was recorded. Memory retrieval was examined 24 h after the learning phase. The mouse was placed in the light compartment (door closed), and 10 s later, the door was opened. The latency to enter the dark chamber (step-through latency; STL) was recorded within 5 min.

#### Conditioned place preference (CPP)

The day after other behavioral assays, CPP was performed to evaluate drug-induced addictive (drug-seeking) behavior^59^. The CPP apparatus was a two-compartment box (30 × 30 × 40 cm), which were connected through a neutral chamber in the middle. One compartment was painted in white with vertical white stripes with a textured floor, and the other was painted in black with horizontal white stripes and a smooth floor. Each room had gates allowing the animal to access all three rooms freely.

Animals were placed in the neutral part of the apparatus on day one and the doors were opened to allow the animal to explore the compartments for 10 min. The time spent in each compartment was recorded and if the animals spent a relatively equal time in the three chambers (not more than 60% of time spent in one compartment), they were included in the study. For CPP conditioning, the mice were injected with saline or morphine (10 mg/kg, i.*p*.) for the next 4 days. The two distinct compartments were paired repeatedly, one with the morphine injections and the other with the saline injections. After the injection of morphine or saline, mice were kept in the respective compartment for 30 min with the doors closed. On the sixth day, animals were again put in the neutral compartment, and all doors were opened with access to all chambers. Time spent in each compartment was recorded, and CPP score was calculated using the following formula:

$$ {\text{CPP }}\;{\text{score }}\left( {{\text{seconds}}} \right)\, = \,{\text{The}}\,{\text{time}}\;{\text{spent}}\;{\text{in}}\;{\text{the}}\;{\text{non - preferred}}\;{\text{chamber }}\;{\text{on}}\;{\text{ post-}}\;{\text{conditioning }}\;{\text{day}} - {\text{The}}\;{\text{time}}\;{\text{spent}}\;{\text{ in}}\;{\text{ the}}\;{\text{non - preferred}}\;{\text{chamber}}\;{\text{on}}\;{\text{ pre-conditioning }}\;{\text{day}} $$

Non-preferred chamber was defined on the first day when the animals spent less than 50% of the time in that compartment.

### Electrophysiological assays

#### Slice preparation

Mice were decapitated under deep anesthesia and the brain was rapidly removed from the skull. All procedures were performed as previously described^33^. The brain was then submerged in artificial cerebrospinal fluid (aCSF) composition containing (in mM) 124 NaCl, 25 NaHCO_3_, 10 d-glucose, 4.4 KCl, 2 MgCl_2_, 1.25 NaH_2_PO_4_ and 2 CaCl_2_. (oxygenated with 5%CO_2_–95%O_2_, pH 7.4 ± 0.05 and osmolarity was adjusted to 300 ± 10 mOsm). The brainstem was then blocked in a coronal plane and sectioned at 250 μm thicknesses on a Vibroslicer (Campden Instrument, NVSLM1, Sarasota, FL, USA). The LDT was identified on the basis of its anatomical location relative to the fourth ventricle. From each animal, two slices including the LDT were selected. Slices containing the LDT were incubated for 45 min at 34 ± 2 °C and then stored at room temperature. The LDT slices were continuously superfused at 1 ml/min with aCSF.

#### Whole-cell patch clamp recording

The effects of PS exposure on the electrophysiological properties of LDT neurons in the male offspring were evaluated by whole-cell patch clamp recordings in LDT brain slices in PND 40–45.

LDT neurons were monitored on a television screen through an infrared charge-coupled device camera (Hamamatsu, Japan) and video microscopy (BX-51, Olympus, Tokyo, Japan). Due to the lack of access to immunohistochemistry and fluorescent microscopy in the intracellular recording laboratory, in order to record the activity of the acetylcholinergic neurons, we first attempted to identify the neurons with the highest physical similarity to acetylcholinergic neurons in the LDT. According to previous studies, cholinergic neurons are characterized by polygonal-shaped large soma, while GABAergic neurons are small round-shaped neurons and glutamatergic neurons are intermittent in size and smaller than cholinergic neurons. Accordingly, neurons with polygonal soma size of larger than 30 mm were selected for whole-cell patch clamp recording^60,61^.

Patch pipettes were filled with internal solution containing (in mM) 140 potassium gluconate, 5 KCl, 10 HEPES, 2 MgCl2, 0.2 EGTA, 2 Na2ATP and 0.4 Na2GTP. The pH and the osmolarity of the internal solution were adjusted to 7.2 (by KOH) and 285 ± 5 mOsm, respectively.

Current clamp whole-cell patch clamp recordings were conducted on LDT neurons as identified by a well-established criteria defined below using a Multiclamp 700B amplifier (Axon Instruments) and signals were digitized by a Digidata 1440 A/D converter (Axon Instruments). Electrophysiological records were filtered at 20 kHz and sampled at 10 kHz. LDT neurons were visualized with a 60 × water immersion objective using Nomarski-type differential interference contrast (DIC) imaging with infrared illumination. After establishment of a giga seal, brief suction was applied to break through the cell membrane for whole-cell configuration*.* Cells with a seal < 1 GΩ before rupture of the membrane were discarded and the test seal function was constantly employed and monitored throughout the recording to ensure that the seal was stable^62^.

### Statistical analysis

Statistical analysis was performed using GraphPad Prism 9.0 software for windows. Kolmogorov–Smirnov test was used to determine the normal distribution of the data. To compare the mean level of variables between the groups while accounting for the impacts of the different types of stressors, one-way ANOVA was employed, followed by Tukey's post-hoc analysis. The time spent in the drug-paired compartment before and after conditioning days was compared using repeated measure ANOVA. *P* < 0.05 was considered statistically significant.

## Ethical approval and informed consent

All procedures performed in this study were in accordance with the ethical standards of the ethical committee of Kerman University of Medical Sciences. All experimental procedures were established in accordance with ARRIVE guidelines and were approved by the Animal Research Ethics Committee of the Kerman Neuroscience Research Center (Ethics code: IR.KMU.REC.1397.579).

## Data Availability

The datasets used or analyzed during the current study are available from the corresponding author on reasonable request.

## Code availability

All software applications used are included in this article.
